# Supplementary material for: Acute regulation of murine adipose tissue lipolysis and insulin resistance by the TGFβ superfamily protein GDF3
Source: Nat Commun. 2025 May 13;16:4432. doi: 10.1038/s41467-025-59673-7 (PMC12075709; doi:10.1038/s41467-025-59673-7)
Supplement: Supplementary file 1 — Supplementary Information [file 41467_2025_59673_MOESM1_ESM.pdf]

Acute regulation of murine adipose tissue lipolysis and insulin resistance by the TGFβ superfamily protein GDF3

Nagasuryaprasad Kotikalapudi[1,#], Deepti Ramachandran[1,#], Daniel Vieira[1], William Bernard Rubio[1], Gregory R. Gipson[2], Luca Troncone[3], Kylie Vestal[2], David E. Maridas[4], Vicki Rosen[4], Paul B.Yu[3], Thomas B. Thompson[2], Alexander S. Banks[1]*

[1]Division of Endocrinology, Diabetes and Metabolism, Beth Israel Deaconess Medical Center and Harvard Medical School, Boston, MA, USA.

[2]Department of Molecular & Cellular Biosciences, University of Cincinnati College of Medicine, Cincinnati, OH, USA.

[3]Cardiovascular Research Center, Massachusetts General Hospital and Harvard Medical School, Boston, MA, USA.

[4]Department of Developmental Biology, Harvard School of Dental Medicine, Boston, MA USA.

[#]These authors contributed equally.

*****corresponding author: asbanks@bidmc.harvard.edu

<u>**SUPPLEMENTARY INFORMATION**</u>

Table S1. Recombinant proteins

Recombinants	Species	Catalog No	Company	Used for
GDF3	Mouse	9009-GD-010	R&D Systems	Flow Cytometry, BLI, ITC, luciferase reporter assay, lipolysis
GDF3	Human	5754-G3-010	R&D Systems	BLI
GDF3	Human	5754-G3-010/CF	R&D Systems	SPR
BMP2	Human	N/A	Vicki Rosen lab	Flow Cytometry, lipolysis
BMP2	Human/ Mouse/ Rat	120-02C-10UG	PeproTech	ITC, lipolysis
BMP4	Mouse	5020-BP-010	R&D Systems	ITC, lipolysis
BMP7	Mouse	5666-BP-010	R&D Systems	Flow Cytometry
BMP9	Human	3209-BP-010	R&D Systems	Flow Cytometry, lipolysis
BMP10	Mouse	6038-BP-025	R&D Systems	Flow Cytometry, lipolysis
TGFβ1	Human	T7039	Millipore Sigma	Flow Cytometry, lipolysis
BMPRII-Fc	Human/ Mouse	811-BR-100	R&D Systems	SPR, BLI, ITC
TGFBRII-Fc	Human	341-BR-050/CF	R&D Systems	SPR
ACTRIIA-Fc	Human	340-R2-100/CF	R&D Systems	SPR
ACTRIIB-Fc	Human	339-RB-100/CF	R&D Systems	SPR
IgG-Fc	Human	160024	Gator Bio	BLI
IgG-Fc	Mouse	160004	Gator Bio	BLI

Table S2. Antibodies

Antibody	Host Species	Catalog No	Company
anti-GDF3	Rabbit [EPR4828]	ab108617	Abcam
anti-β-actin	Mouse	A1978	Sigma
anti-Phospho-HSL	Rabbit	45804	Cell Signaling Technology
anti-HSL	Rabbit	18381	Cell Signaling Technology
anti-β3-AR	Rabbit	PA5-50914	Invitrogen
anti-cAMP	Mouse [250532]	MAB2146	R&D Systems
anti-rabbit IgG, HRP-linked	Goat	7074	Cell Signaling Technology
anti-mouse IgG, HRP-linked	Horse	7076	Cell Signaling Technology
anti-mouse IgG (Alexa 488)	Goat	ab150117	Abcam
anti-rabbit IgG (Alexa 647)	Goat	A21244	Life Technologies

Table S3: **qPCR primer list**

Murine Gene Name	Forward Primer (5' to 3')	Reverse Primer (5' to 3')
Gdf3 (growth differentiation factor 3)	CTTCTCCCAGACCAGGGTTTT	TCTAGAGTCAGCTGGGCCAT
Gdf3 (codon optimized inTetOn-mGdf3 plasmid)	GGTACAGCAGGGTGACAGTG	GCAAAATCCCTTAGGCACGG
Tbp (TATA-box binding protein)	CCCTATCACTCCTGCCACACCAGC	GTGCAATGGTCTTTAGGTCAAGTTTACAGCC
Id1 (inhibitor Of DNA Binding 1)	CCTAGCTGTTCGCTGAAGGC	CTCCGACAGACCAAGTACCAC
Id2 (inhibitor Of DNA Binding 2)	ATGAAAGCCTTCAGTCCGGTG	AGCAGACTCATCGGGTCGT
Id4 (inhibitor Of DNA Binding 4)	CAGTGCGATATGAACGACTGC	GACTTTCTTGTTGGGCGGGAT
Lamb3 (laminin subunit Beta 3)	GGCTGCCTCGAAATTACAACA	ACCCTCCATGTCTTGCCAAAG
Zbtb16 (zinc finger and BTB domain containing 16)	CTGGGACTTTGTGCGATGTG	CGGTGGAAGAGGATCTCAAACA
Snai2 (snail family transcriptional repressor 2)	TGGTCAAGAAACATTTCAACGCC	GGTGAGGATCTCTGGTTTTGGTA
Slpi (secretory leukocyte peptidase inhibitor)	GGCCTTTTACCTTTCACGGTG	TACGGCATTGTGGCTTCTCAA
Hdac4 (histone deacetylase 4)	CTGCAAGTGGCCCCTACAG	CTGCTCATGTTGACGCTGGA
Adamts4 (ADAM metallopeptidase with thrombospondin type 1 motif 4)	ATGGCCTCAATCCATCCCAG	AAGCAGGGTTGGAATCTTTGC
Adamts5 (ADAM metallopeptidase with thrombospondin type 1 motif 5)	GGAGCGAGGCCATTTACAAC	CGTAGACAAGGTAGCCCACTTT

Adamts7 (ADAM metallopeptidase with thrombospondin type 1 motif 7)	AGTGTCCCAACCTGCCATTTG	CCTAGAGCCTTGGTGCTTGTA
Col3a1 (collagen type III alpha 1 chain)	CTGTAACATGGAAACTGGGGAAA	CCATAGCTGAACTGAAAACCACC
Col5a3 (collagen type V alpha 3 chain)	CGGGGTACTCCTGGTCCTAC	GCATCCCTACTTCCCCCTTG
Col18a1 (collagen type XVIII alpha 1 chain)	GTGCCCATCGTCAACCTGAA	GACATCTCTGCCGTCAAAAGAA
Col27a1 (collagen type XXVII alpha 1 chain)	CCTTCCCGTAGGGACTCCAT	GGCACAGTAATTGTGAGCGAC
Adrb3 (adrenergic receptor beta 3)	GGCCCTCTCTAGTTCCCAG	TAGCCATCAAACCTGTTGAGC
Pparγ (Peroxisome proliferator-activated receptor gamma	GCATGGTGCCTTCGCTGA	TGGCATCTCTGTGTCAACCATG
AdipoQ (Adiponectin)	TGTTCCTCTTAATCCTGCCCA	CCAACCTGCACAAGTTCCCTT
Leptin	GAGACCCCTGTGTCGGTTC	CTGCGTGTGTGAAATGTCATTG
Perf-1 (Preadipocyte factor 1)	CCTGGGTTCTCTGGAAAGGACTG	TGGTTGCGGCTACGATCTCAC

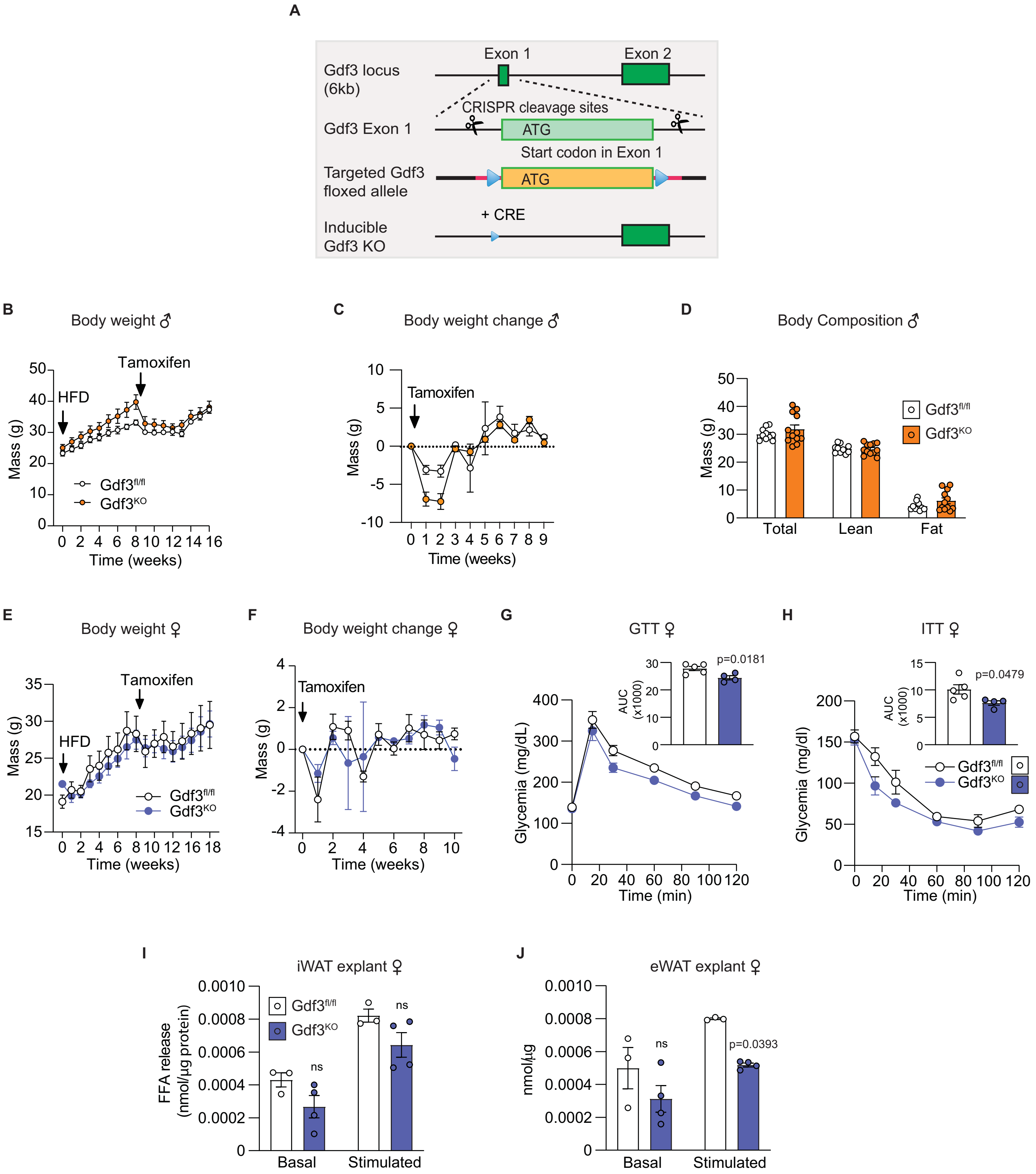

Supplementary Figure 1

A) Graphical representation of the Gdf3 locus where CRISPR cas9 sites were integrated to span exon 1, such that upon Cre recombinase activity, exon 1 of Gdf3 would be deleted, leading to GDF3 KO mice or cells. B) Body weights of adult male $Gdf3^{fl/fl}$ and $Gdf3^{fl/fl}$::Rosa-$Cre^{ERT2/-}$ mice over time from the onset of HFD feeding and during and after tamoxifen injections to generate $Gdf3^{fl/fl}$ control and $Gdf3^{KO}$ mice (n =9/10). C) Body weight change after tamoxifen injections in mice in (B). D) Body composition of mice in (B) at week 14. B-D) n = 10 $Gdf3^{fl/fl}$, 11 $Gdf3^{KO}$. E-J) Adult female $Gdf3^{fl/fl}$ and $Gdf3^{fl/fl}$ x Rosa-$Cre^{ERT2/-}$ mice, fed HFD for 8 weeks followed by tamoxifen injections to generate $Gdf3^{fl/fl}$ control and $Gdf3^{KO}$ mice (n =4). E) Body weights from the onset of HFD feeding. F) Body weight change after tamoxifen injections in mice in (E). G) Left: GTT, 4 weeks post tamoxifen injections (glucose dose: 1g/kg BW, IP). Right inset: AUC of GTT. H) Left: ITT, 5 weeks post tamoxifen injections (insulin dose: 0.75U/kg BW, IP). Right inset: AUC of ITT. E-H) n = 5 $Gdf3^{fl/fl}$, 4 $Gdf3^{KO}$. I-J) FFA release from tissue explants at baseline and following lipolytic stimulation with isoproterenol (n = 3 $Gdf3^{fl/fl}$, 4 $Gdf3^{KO}$). I) iWAT, J) eWAT. Statistical comparisons were made using two way ANOVA with Sidak's multiple comparisons (B, C, E, F, I, J), unpaired two-tailed Student's t-test (D, G, H). B-J) Data are presented as mean values +/- SEM, ns = not significant. Source data are provided as a Source Data file.

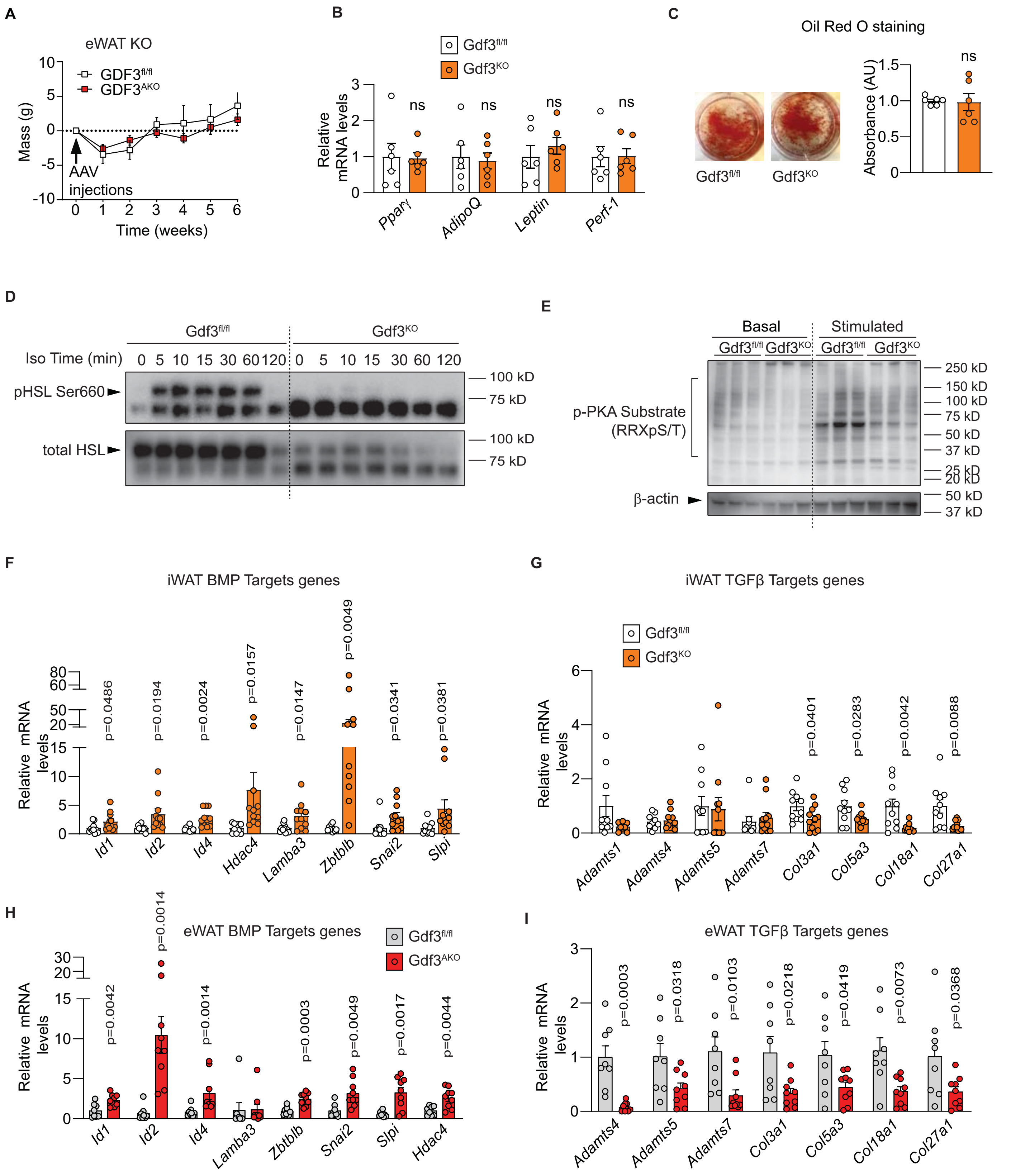

Supplementary Figure 2

A) Body weight changes after AAV injections in mice from Figure 2 (n = 8 Gdf3$^{fl/fl}$, 9 Gdf3^{AKO}). B) RT-qPCR analysis of adipogenic genes in cultured primary Gdf3$^{fl/fl}$ and Gdf3^{KO} adipocytes (n = 6). C) Left: Representive images of oil red O staining of cultured primary Gdf3$^{fl/fl}$ and Gdf3^{KO} adipocytes. Right: Quantification of oil red O from stained cultured primary Gdf3$^{fl/fl}$ and Gdf3^{KO} adipocytes (n = 6). D-E) Western blot analysis following lipolytic stimulation with isoproterenol for indicated time points. D) Phosphorylated hormone-sensitive lipase at serine 660 (pHSL Ser 660) and corresponding total HSL. E) Phosphorylated protein kinase A (p-PKA) substrates prior to (basal) and after 15 minutes of isoproterenol (Stimulated). β-actin levels serve as the loading control (n = 3 biological replicates per group). F-G) RT-qPCR analysis of genes in iWAT of Gdf3$^{fl/fl}$ and Gdf3^{KO} mice from Figure 1. F) BMP signaling targets (n = 10 Gdf3$^{fl/fl}$, 11 Gdf3^{KO}). G) TGFβ signaling targets (n = 10 Gdf3$^{fl/fl}$, 11 Gdf3^{KO} except for *Adamts4* and *Adamts7* where n = 9 Gdf3$^{fl/fl}$, 11 Gdf3^{KO}). H-I) RT-qPCR analysis of genes in eWAT of Gdf3$^{fl/fl}$ and Gdf3^{AKO} mice from Figure 2 (n = 8 Gdf3$^{fl/fl}$, 9 Gdf3^{AKO}). H) BMP signaling targets. **I**) TGFβ signaling targets. A-C, F-I) Statistical comparisons were made using two way ANOVA with Sidak's multiple comparisons (A), unpaired two-tailed Student's t-test (B, C, F-I). A-C, F-I) Data are presented as mean values +/- SEM, ns = not significant. Source data are provided as a Source Data file.

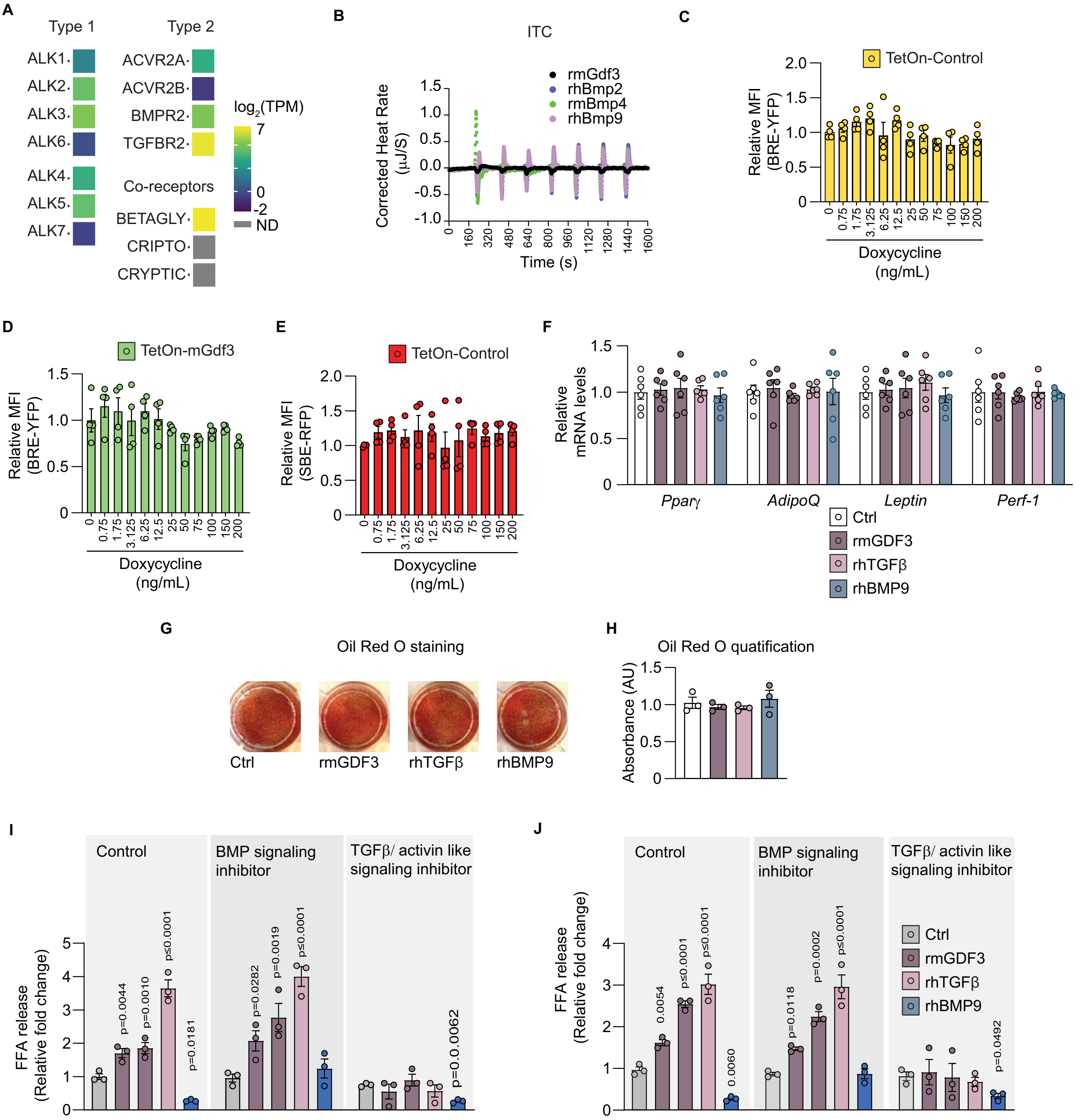

Supplementary Figure 3

A) mRNA expression levels of indicated receptors and coreceptors from RNA seq data set of immortalized murine differentiated adipocytes (E-MTAB-2624 from ArrayExpress as described in Shinoda et al. Nat Med. 2015 Apr;21(4):389-94. doi: 10.1038/nm.3819). B) Isothermal Titration Calorimetry (ITC) assay showing the binding isotherm for the titration of 250ng/mL rhBMP2, rmBMP4 or rhBMP9 against 250ng/mL of rhGDF3. C-E) C2C12 dual reporter myoblasts were transiently transfected with TetOn-Control or TetOn-mGdf3 vectors as indicated for 24 hours, followed by doxycycline treatment at increasing doses in serum-free media for 48 hours. Cells expressing YFP, RFP, and BFP were sorted via flow cytometry and analyzed for changes in reporter activity (n = 4 biological replicates, where each n represents the average MFI of all triple positive live cells per well). C) Relative MFI of BRE-YFP in cells transfected with TetOn-Control. D) Relative MFI of BRE-YFP in cells transfected with TetOn-mGdf3. E) Relative MFI of SBE-RFP in cells transfected with TetOn-Control. F-H) Immortalized murine iWAT SVF cells were cultured and differentiated into mature adipocytes followed by treatment with the indicated recombinant proteins under serum free conditions. Recombinant doses: rmGdf3 (500 and 1000 ng/mL); rhTGFβ1 (5 ng/mL); rhBMP9 (125 ng/mL). F) RT-qPCR analysis of adipogenic genes (n = 6). G) Representative images of Oil red O staining. H) Quantification of oil red O staining (n = 3). I-J) Relative free fatty acid release from cultured adipocytes serum starved for three hours, pretreated with serum free media alone (Control) or LDN193189 (BMP signaling inhibitor, 0.5 μM) or SB-431542 (TGFβ/activin-like signaling inhibitor, 5 μM) for 1 hour, followed by 24 hours of incubations with recombinant proteins prior to beta adrenergic stimulation with isoproterenol for two hours. Recombinant doses: rmGDF3 (500, 1000 ng/mL); rhTGFβ1 (2.5 ng/mL); rhBMP2 (63 ng/mL) (I) or rmBMP4 (63 ng/mL) (J); (n = 3). C-F, H-J) Statistical comparisons were made using one way ANOVA with Dunnett's multiple comparisons test. C-F, H-J) Data are presented as mean values +/- SEM, ns = not significant. Source data are provided as a Source Data file.

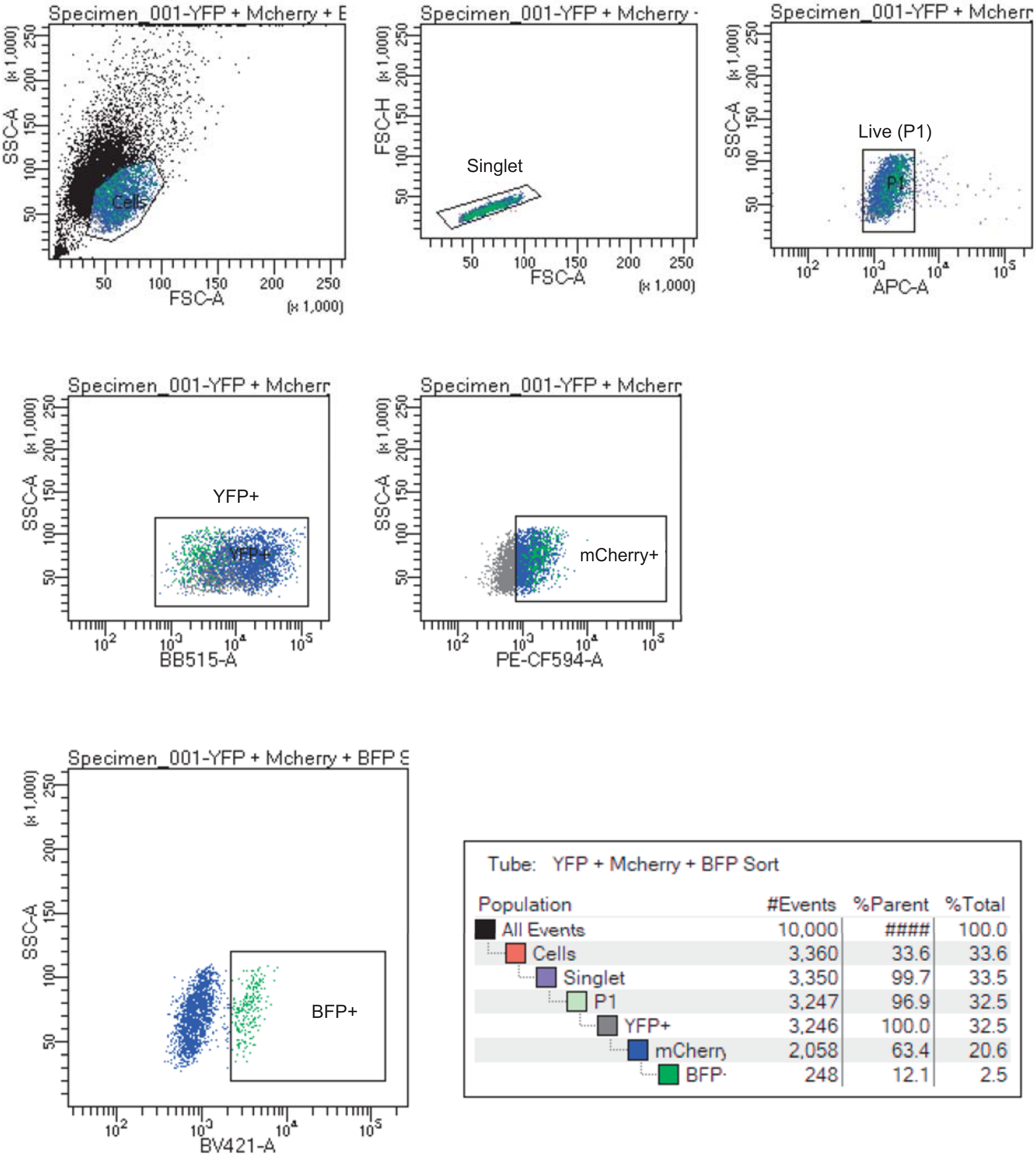

Tube: YFP + Mcherry + BFP Sort

Population	#Events	%Parent	%Total
All Events	10,000	####	100.0
Cells	3,360	33.6	33.6
Singlet	3,350	99.7	33.5
P1	3,247	96.9	32.5
YFP+	3,246	100.0	32.5
mCherry	2,058	63.4	20.6
BFP	248	12.1	2.5

Supplementary Figure 4

Representative gating strategy for flow cytometry analysis. C2C12 dual reporter cells (YFP and mCherry double positive cells), were transiently transfected with a vector expressing doxycycline inducible TetOnControl or TetOn-Gdf3 (expressing BFP). Cells triple positive for YFP, RFP (mCherry) and BFP were sorted for analysis and the mean fluorescence intensities (MFI) of YFP and RFP of these cells were plotted for analysis. Similarly, for the double positive preadipocyte reporter cells, YFP and RFP double positive cells were sorted for analysis of MFI's of YFP and RFP.
