# Supplementary material for: Acute regulation of murine adipose tissue lipolysis and insulin resistance by the TGFβ superfamily protein GDF3
Source: Nat Commun. 2025 May 13;16:4432. doi: 10.1038/s41467-025-59673-7 (PMC12075709; doi:10.1038/s41467-025-59673-7)
Supplement: Supplementary file 2 — Reporting Summary [file 41467_2025_59673_MOESM2_ESM.pdf]

nature portfolio

Corresponding author(s): Alexander S Banks

Last updated by author(s): Apr 7, 2025

Reporting Summary

Nature Portfolio wishes to improve the reproducibility of the work that we publish. This form provides structure for consistency and transparency in reporting. For further information on Nature Portfolio policies, see our Editorial Policies and the Editorial Policy Checklist.

Statistics

For all statistical analyses, confirm that the following items are present in the figure legend, table legend, main text, or Methods section.

n/a	Confirmed	
☐	☒	The exact sample size (*n*) for each experimental group/condition, given as a discrete number and unit of measurement
☐	☒	A statement on whether measurements were taken from distinct samples or whether the same sample was measured repeatedly
☐	☒	The statistical test(s) used AND whether they are one- or two-sided<br>*Only common tests should be described solely by name; describe more complex techniques in the Methods section.*
☐	☒	A description of all covariates tested
☐	☒	A description of any assumptions or corrections, such as tests of normality and adjustment for multiple comparisons
☐	☒	A full description of the statistical parameters including central tendency (e.g. means) or other basic estimates (e.g. regression coefficient) AND variation (e.g. standard deviation) or associated estimates of uncertainty (e.g. confidence intervals)
☐	☒	For null hypothesis testing, the test statistic (e.g. *F*, *t*, *r*) with confidence intervals, effect sizes, degrees of freedom and *P* value noted<br>*Give P values as exact values whenever suitable.*
☒	☐	For Bayesian analysis, information on the choice of priors and Markov chain Monte Carlo settings
☒	☐	For hierarchical and complex designs, identification of the appropriate level for tests and full reporting of outcomes
☒	☐	Estimates of effect sizes (e.g. Cohen's *d*, Pearson's *r*), indicating how they were calculated

Our web collection on statistics for biologists contains articles on many of the points above.

Software and code

Policy information about availability of computer code

Data collection	Promethion Indirect calorimeter (Sable Systems International)<br>Telemetry CGM probes (DSI)<br>Body Composition Analyzer EchoMRI (Echo Medical Systems)<br>QuantStudio 6 Real-Time PCR System (Applied Biosystems)<br>EPOCH 2 Plate Reader (BioTek)<br>BioTek Synergy H1 hybrid plate reader (BioTek)<br>ChemiDoc Touch Imaging System (Biorad)<br>Nano ITC Standard Volume (TA Instruments)<br>CytoFLEX flow Cytometer (Beckman Coulter)<br>Benchling [Biology Software] (2021-2024)<br>Zeiss Confocal System (Zeiss)<br>Zeiss V8 Stereoscope (Zeiss)<br>Biacore T200 optical biosensor system (Cytiva)<br>Gator Plus instrument (Gator Bio)
Data analysis	Image lab touch software version 3.0.1, Cytoflex, Image J software 1.52a, CytExpert2.3, GraphPad Prism Version 8, R version 4.3.0, Iglu package version 3.4.2, tidyverse package, CalR version 1.3, Nano Analyze Software, Fiji software 1.54f, Biacore software, Gator Bio software

For manuscripts utilizing custom algorithms or software that are central to the research but not yet described in published literature, software must be made available to editors and reviewers. We strongly encourage code deposition in a community repository (e.g. GitHub). See the Nature Portfolio guidelines for submitting code & software for further information.

Data

Policy information about availability of data

All manuscripts must include a data availability statement. This statement should provide the following information, where applicable:

- Accession codes, unique identifiers, or web links for publicly available datasets
- A description of any restrictions on data availability
- For clinical datasets or third party data, please ensure that the statement adheres to our policy

All data generated in this study are provided within the paper or in the Supplementary information or the Source data file unless specified otherwise. Large data sets from indirect calorimetry, isothermal calorimetry, surface plasmon resonance and biolayer interferometry do not have a suitable public repository for deposition and will therefore be made available upon request.

Research involving human participants, their data, or biological material

Policy information about studies with human participants or human data. See also policy information about sex, gender (identity/presentation), and sexual orientation and race, ethnicity and racism.

Reporting on sex and gender	Not applicable (no human data)
Reporting on race, ethnicity, or other socially relevant groupings	Not applicable (no human data)
Population characteristics	Not applicable (no human data)
Recruitment	Not applicable (no human data)
Ethics oversight	Not applicable (no human data)

Note that full information on the approval of the study protocol must also be provided in the manuscript.

Field-specific reporting

Please select the one below that is the best fit for your research. If you are not sure, read the appropriate sections before making your selection.

[x] Life sciences [ ] Behavioural & social sciences [ ] Ecological, evolutionary & environmental sciences

For a reference copy of the document with all sections, see nature.com/documents/nr-reporting-summary-flat.pdf

Life sciences study design

All studies must disclose on these points even when the disclosure is negative.

Sample size	No sample size calculations were performed, sample size was determined by the availability of mouse samples.
Data exclusions	No data exclusions.
Replication	The experimental findings were reliably reproduced as validated by at least two independent experiments except for the continuous glucose monitoring experiments
Randomization	Samples/animals were randomly allocated to experimental groups and processed in all experiments.
Blinding	Blinding was not possible in experiments because cells or mice needed to be genotyped and all experiments were run by staff who were aware of the experimental groups.

Reporting for specific materials, systems and methods

We require information from authors about some types of materials, experimental systems and methods used in many studies. Here, indicate whether each material, system or method listed is relevant to your study. If you are not sure if a list item applies to your research, read the appropriate section before selecting a response.

Materials & experimental systems

n/a	Involved in the study
☐	☒ Antibodies
☐	☒ Eukaryotic cell lines
☒	☐ Palaeontology and archaeology
☐	☒ Animals and other organisms
☒	☐ Clinical data
☒	☐ Dual use research of concern
☒	☐ Plants

Methods

n/a	Involved in the study
☒	☐ ChIP-seq
☐	☒ Flow cytometry
☒	☐ MRI-based neuroimaging

Antibodies

Antibodies used

anti-GDF3 Rabbit [EPR4828] ab108617 Abcam (1:1000)
anti-β-actin Mouse A1978 Sigma (1:2000)
anti-Phospho-HSL Rabbit 45804 Cell Signaling Technology (1:1000)
anti-HSL Rabbit 18381 Cell Signaling Technology (1:1000)
anti-β3-AR Rabbit PA5-50914 Invitrogen (1:100 for confocal) and (1:400 for western blot)
anti-cAMP Mouse [250532] MAB2146 R&D Systems (1:100)
anti-rabbit IgG, HRP-linked Goat 7074 Cell Signaling Technology (1:10,000)
anti-mouse IgG, HRP-linked Horse 7076 Cell Signaling Technology (1:10,000)
anti-mouse IgG (Alexa 488) Goat ab150117 Abcam (1:500)
anti-rabbit IgG (Alexa 647) Goat A21244 Life Technologies (1:500)

Validation

anti-GDF3 Rabbit [EPR4828] ab108617 Abcam - validated using doxycycline induced mouse GDF3 protein expression. C2C12 cells have no endogenous expression of Gdf3 and cells transfected with doxycycline induced BFP were used as negative control. Also no protein expression was seen in cells transfected with either control of Gdf3 plasmids in the absence of doxycycline.

All other antibodies for indicated use in our paper are fully validated on the manufacture's website.
anti-β-actin Mouse A1978 Sigma (https://www.sigmaaldrich.com/US/en/product/sigma/a1978)
anti-Phospho-HSL Rabbit 45804 Cell Signaling Technology (https://www.cellsignal.com/products/primary-antibodies/phospho-hsl-ser660-antibody/45804)
anti-HSL Rabbit 18381 Cell Signaling Technology (https://www.cellsignal.cn/products/primary-antibodies/hsl-d6w5s-xp-174-rabbit-mab/18381)
anti-β3-AR Rabbit PA5-50914 Invitrogen (https://www.thermofisher.com/antibody/product/beta-3-Adrenergic-Receptor-Antibody-Polyclonal/PA5-50914)
anti-cAMP Mouse [250532] MAB2146 R&D Systems (https://www.rndsystems.com/products/camp-antibody-250532_mab2146)
anti-rabbit IgG, HRP-linked Goat 7074 Cell Signaling Technology (https://www.cellsignal.com/products/secondary-antibodies/anti-rabbit-igg-hrp-linked-antibody/7074)
anti-mouse IgG, HRP-linked Horse 7076 Cell Signaling Technology (https://www.cellsignal.com/products/secondary-antibodies/anti-mouse-igg-hrp-linked-antibody/7076)
anti-mouse IgG (Alexa 488) Goat ab150117 Abcam (https://www.abcam.com/en-pk/products/secondary-antibodies/goat-mouse-igg-h-l-alexa-fluor-488-preadsorbed-ab150117#)

Eukaryotic cell lines

Policy information about cell lines and Sex and Gender in Research

Cell line source(s)

HEK293T cells (human female in origin)
C2C12 myoblasts (ECACC, # 91031101, C3H murine female in origin)
Immortalized mouse iWAT-SVF cells from Shingo Kajimura's lab (Shinoda, K. et al. Nature medicine, 2015)

Authentication

None of the cell lines were authenticated

Mycoplasma contamination

None of the cell lines were tested for mycoplasma contamination

Commonly misidentified lines
(See ICLAC register)

No commonly misidentified lines were used in this study

Animals and other research organisms

Policy information about studies involving animals; ARRIVE guidelines recommended for reporting animal research, and Sex and Gender in Research

Laboratory animals

Gdf3fl/fl mice generated in C57Bl6/N background but backcrossed to C57Bl/6J for 6 generations.

	Rosa-CreERT2/- mice (B6.129-Gt(ROSA)26Sortm1(cre/ERT2)Tyj/J, The Jackson Laboratory, Stock # 008463)<br>All experiments were initiated in adult 8-week old male or female Gdf3fl/fl and Gdf3fl/fl::Rosa-CreERT2/- mice<br>Primary SVF isolations from Gdf3fl/fl male or female mice was done between 6 to 12 weeks of age.
Wild animals	This study did not involve the use of wild animals
Reporting on sex	Whole body Gdf3 KO studies were done independently in both male and female cohorts. Sex was not considered as a statistical variable as these cohorts were studied independent of each other but both sexes showed similar responses to loss of function of GDF3. Sex of mice was determined prior to weaning using genitalia identification. Sex, age and numbers of mice used in each experiment are mentioned in the figure legends.
Field-collected samples	No field samples were collected in this study.
Ethics oversight	All animal experiments were performed with approval from the Institutional Animal Care and Use Committees (IACUC) of The Harvard Center for Comparative Medicine, Beth Israel Deaconess Medical Center, Boston, USA.

Note that full information on the approval of the study protocol must also be provided in the manuscript.

Plants

Seed stocks	*Report on the source of all seed stocks or other plant material used. If applicable, state the seed stock centre and catalogue number. If plant specimens were collected from the field, describe the collection location, date and sampling procedures.*
Novel plant genotypes	*Describe the methods by which all novel plant genotypes were produced. This includes those generated by transgenic approaches, gene editing, chemical/radiation-based mutagenesis and hybridization. For transgenic lines, describe the transformation method, the number of independent lines analyzed and the generation upon which experiments were performed. For gene-edited lines, describe the editor used, the endogenous sequence targeted for editing, the targeting guide RNA sequence (if applicable) and how the editor was applied.*
Authentication	*Describe any authentication procedures for each seed stock used or novel genotype generated. Describe any experiments used to assess the effect of a mutation and, where applicable, how potential secondary effects (e.g. second site T-DNA insertions, mosiacism, off-target gene editing) were examined.*

Flow Cytometry

Plots

Confirm that:

- [x] The axis labels state the marker and fluorochrome used (e.g. CD4-FITC).
- [x] The axis scales are clearly visible. Include numbers along axes only for bottom left plot of group (a 'group' is an analysis of identical markers).
- [ ] All plots are contour plots with outliers or pseudocolor plots.
- [x] A numerical value for number of cells or percentage (with statistics) is provided.

Methodology

Sample preparation	Immortalized iWAT-SVF preadipocytes or C2C12 myoblasts were cotransfected with the BRE-YFP reporter plasmid and the piggyback transposase (p-base plasmid) designed to be stably integrated using the piggybac transposon system 77. Stable cells were generated by selection with 500µg/mL or 800µg/mL of Hygromycin B (Sigma-Aldrich, # H7772) respectively. The cells were then co-transfected with the SBE-RFP reporter and the p-base plasmid. Cells were selected for stable integration of both reporters by two rounds of fluorescence-assisted cell sorting for YFP and RFP double-positive cells (dual reporter cells). Immortalized preadipocyte dual reporter cells or C2C12 dual reporter cell assays were all performed in 96 well cell culture plates. Briefly after the experimental interventions, the 96 well plate media was removed, and cells were washed with PBS. Cells were then dissociated with 0.25% Trypsin EDTA in a cell culture incubator for approximately 5 minutes. Trypsin was neutralized with PBS containing 3% iFBS, and the plate was spun in a refrigerated centrifuge at 500g for 10 minutes with a swinging rotor. The supernatant was dumped, and FACS buffer (PBS + 0.3% BSA) containing 1:10000 LIVE/DEAD Fixable Far Red Dead Cell Stain (Thermo Fisher Scientific, # L34973) was added to the cells for 15 minutes on ice. The plate was then centrifuged again; the media was removed, washed with FACS buffer, and centrifuged again. The supernatant was removed, and each well was incubated with 200uL of FACS buffer.
Instrument	CytoFLEX flow Cytometer (Beckman Coulter)
Software	CytExpert2.3
Cell population abundance	We performed 96 well plate flow sort assays with atleast 1000 live cells where greater than 95% of cells were positive for both the stably integrated YFP and RFP reporters.

Gating strategy	Briefly, single unstained cells and single fluorescent controls were used to set the gains and gates for each fluorescent channel. Cells were gated to analyze atleast 1000 live single cells from each well. The mean fluorescence intensity (MFI) of either YFP or RFP of the live cells from each well was used for the subsequent analysis. For the doxycycline-induced BFP or GDF3 and BFP expression experiments, the MFI of YFP or RFP was measured from cells expressing YFP, RFP, and BFP.

☒ Tick this box to confirm that a figure exemplifying the gating strategy is provided in the Supplementary Information.
